# Retrospective analysis of the clinical effect of spinal cord stimulation in the treatment of painful diabetic peripheral neuropathy

**DOI:** 10.3389/fneur.2025.1619851

**Published:** 2025-08-21

**Authors:** Dongqiang Cui, Ming Yang, Yang Qiao, Zhenxing Gong, Zeqing Hu, Zhuang Ma, Yang Wu, Guitong Huo

**Affiliations:** ^1^Department of Neurosurgery, Xingtai Ninth Hospital, Xingtai, China; ^2^Department of Neurosurgery, The Second Hospital of Hebei Medical University, Shijiazhuang, China

**Keywords:** diabetic peripheral neuropathy, spinal cord stimulation, lower limb pain, neuromodulation, painful diabetic peripheral neuropathy

## Abstract

**Introduction:**

The aim of this study was to evaluate the clinical outcomes of spinal cord stimulation (SCS) in patients with painful diabetic peripheral neuropathy (PDPN).

**Materials and methods:**

Ninety-two patients underwent permanent SCS implantation and completed a 6-month post-operative follow-up. The primary endpoint was patient amputation rate, and secondary endpoints included Quality of Life (QOL LC V2.0) scale, pain visual analogue scale (VAS), limb nerve conduction velocity, latency and amplitude, and vibration perception threshold (VPT).

**Results:**

In patients with diabetic peripheral neuropathy, QOL LC V2.0 and VAS scores were significantly improved at 6 months postoperatively compared to preoperatively (24.74%, *p* < 0.05; 71.87%, *p* < 0.05). Compared with the median and ulnar nerves of the upper extremity peripheral nerves, the conduction velocities of the common peroneal, sural, superficial peroneal and tibial nerves of the lower extremity peripheral nerves were increased (*p* < 0.05), the latency was significantly shortened (*p* < 0.05) and the amplitude was significantly increased (*p* < 0.05) at 6 months after surgery. Compared to the dorsum of the hand, VPTs were significantly lower in the first toe and dorsum of the foot at 6 months postoperatively compared to preoperatively (*p* < 0.05). The overall amputation rate was 8.69%, with 3.26% for major amputation (above the ankle) and 5.43% for minor amputation (below the ankle).

**Conclusion:**

SCS can effectively relieve lower limb pain in patients with diabetic peripheral neuropathy, repair lower limb peripheral nerves, improve patients’ quality of life, and reduce amputation rate.

## Introduction

Diabetes causes systemic damage, has a profound impact on health-related quality of life and can be life-threatening (1, 2). Diabetic peripheral neuropathy (PDN) is a common complication that manifests as pain and other sensory disturbances, including numbness, burning or tingling. About 20% of people with diabetes develop painful diabetic neuropathy (3, 4). Diabetic foot and lower limb amputation is a common complication of PDN, which has a devastating impact on the health of patients and their families, and is expensive and difficult to treat. According to statistics, the overall amputation rate of diabetic foot patients in China is 19.03%, with a major amputation rate of 2.14% and a minor amputation rate of 16.88% (5). Although there are a number of treatments for PDN, randomised clinical trials have shown that these drugs have limited efficacy and a high incidence of adverse events (6–10). Currently, the treatment of diabetic foot remains a challenging problem.

Spinal cord stimulation (SCS) is an invasive neurosurgical procedure. The use of SCS devices for the treatment of lower extremity pain has been reported since the 1970s (11). The device activates the thick Aβ fibres in the dorsal columns of the spinal cord. This excitation inhibits the synaptic transmission of pain-related C fibres (nociceptive) and Aδ fibres (allodynia) in the dorsal horn of the spinal cord, thereby closing the ‘pain gate’ (12). Previous studies have shown that high-frequency (10 kHz) SCS can significantly relieve neuropathic pain in the extremities while reducing opioid doses (13–22). Low-frequency (40–60 Hz) SCS has been shown to be of moderate benefit in two previous randomised controlled trials (23–25). A previous single-centre study showed that 40 Hz SCS is effective in improving the clinical symptoms of DPN. A cohort study of SCS and endovascular revascularisation was also reviewed (26–28). Due to the small sample size of the previous study, we conducted this retrospective study to confirm the clinical efficacy of SCS in treating PDPN, particularly with regard to nerve repair. This was achieved by comparing and analysing patients’ postoperative limb nerve conduction velocity, latency and amplitude, as well as their vibration perception threshold (VPT).

## Materials and methods

### Research design

We assessed VAS, QOL, and limb nerve conduction (velocity, latency, amplitude) and vibration perception threshold (VPT) in patients with diabetic peripheral neuropathy before SCS using a single-blind questionnaire (Collectors (e.g., nurses or laboratory assistants) were unaware of the participants’ subgroups and only provided standardised questionnaires to be completed by the participants to reduce assessment bias.) and electromyography equipment, and followed up 6 months after surgery by telephone and outpatient re-examination. To reduce information bias, two doctors used a blinded method to collect patient data and repeated measurements to reduce random error. The changes in VAS and QOL were compared between preoperative and 6 months postoperative, and the clinical remission rate was calculated; the effects of SCS on the peripheral nerves of the lower limbs (common peroneal, peroneal, superficial peroneal, and tibial nerves) were analysed, with the peripheral nerves of the upper limbs (including the ulnar nerve and median nerve) set up as a control group; Changes in vibration perception threshold of the first toe, dorsum of the foot and dorsum of the hand were simultaneously compared between preoperative and 6 months postoperative, and the final statistics of amputation rate were made at 6 months postoperative. This study was approved by the Ethics Committee of Xingtai Ninth Hospital.

### Participants

Patients in this study were recruited from a neurosurgery clinic between January 2020 and January 2025. After receiving a full description of the procedure and associated risks, all patients gave written informed consent to participate in the study and follow-up. All studies reported here were conducted in accordance with the Declaration of Helsinki.

The inclusion criteria for the study were as follows: (1) age≥18 years, diagnosed with painful diabetic peripheral neuropathy; (2) pain visual analysis scale (VAS) ≥ 5 cm; (3) patient’s physical condition is generally good and can tolerate surgery. Exclusion criteria were as follows: (1) patients with neurosurgical contraindications or inability to tolerate surgical treatment; (2) pregnant and lactating women; (3) expected life expectancy < 2 years; (4) patients refusing to participate in follow-up.

### Surgical procedures

In the first step, the patient was placed in the prone position and the Medtronic SPECIFY 565 electrode (US brand) was placed outside the T10-T12 dura mater under local anaesthesia. Paresthesia-base stimulation (40 Hz, 180–240 μs. 0.5–2.0 V) was then performed. Patients entered the permanent implantation phase if they experienced ≥50% pain relief (decrease in VAS score) and did not experience any serious adverse events (e.g., infection or electrode displacement) during the trial phase. In the second stage, 1 week after the stimulation test, the previous incision is reopened and the implanted leads are connected to the implantable pulse generator (IPG) using connector leads.

### Postoperative care

All patients with a diabetic foot ulcer were treated with standardised foot debridement, including debridement of infected and necrotic tissue until the wound was clean (29, 30). At the same time, appropriate antibiotics were selected based on the results of secretion culture and drug susceptibility testing.

### Clinical evaluation

#### Visual analog scale

The Visual Analogue Scale is the most commonly used pain rating scale, where the level of pain is rated from 0 to 10 cm, with 0 cm being no pain and 10 cm being the most severe pain (31).

#### Quality of life

The Quality of Life Scale (QOL-LC V2.0) is widely used to measure the physical and mental health of patients with chronic conditions (32). It is a questionnaire consisting of 23 questions (with a maximum score of 320), and a higher score indicates a higher quality of life.

#### Nerve conduction velocity, latency and amplitude

Electromyography is used to monitor the recovery process and effectiveness of peripheral neuropathy conditions during the treatment process (33). The electrode is attached to a fixed position on the skin of the limb to detect bioelectric signals in resting or contracting surface muscles. Nerve conduction velocity is measured in metres per second (m/s) and a slow conduction velocity indicates the severity of nerve damage. Latency is measured in milliseconds (ms), and a long latency indicates the severity of the injury. Motor nerve amplitude is measured in millivolts (mv), while sensory nerve amplitude is measured in microvolts (μv). A large amplitude indicates good nerve function (34).

#### Vibration perception threshold

Vibration Perception Threshold (VPT) Defined as the lowest voltage at which vibration can be detected. VPT has been shown to be a good predictor and prognosticator of long-term complications in diabetic peripheral neuropathy (DPN) (35). VPT is measured in volts (v), with a higher value indicating poorer vibration perception.

#### Amputation rate

A major amputation is defined as an amputation above the ankle joint, while a minor amputation is defined as an amputation below the ankle joint.

### Data analysis

GraphPad Prism statistical software was used for data analysis and image processing. Continuous variables are presented as mean ± standard deviation and categorical variables as percentages. Paired t-tests were used to compare variables before and 6 months after surgery. *p* < 0.05 was considered significant.

## Results

### Demographic data

Between 1 January 2018 and 1 January 2023, we recruited 92 patients with diabetic foot peripheral neuropathy from the neurosurgery clinic and performed a 6-month postoperative follow-up. Demographic characteristics are shown in Table 1.

**Table 1:** Demographic characteristics of DPN patients.

Variable (*n* = 92)	Preoperative*	Postoperative*	*T*	*p* value
Age, y	67.48 ± 11.35	67.98 ± 11.35	–	–
Male, *n* (%)	65 (70.7%)	65 (70.7%)	–	–
BMI, kg/m^2^	24.38 ± 1.21	23.96	4.83	0.527
Glycated Hemoglobin (HbA1C)%	8.37 ± 0.91	8.19 ± 0.85	2.67	0.658
Duration of diabetes, years	17.43 ± 10.68	17.93 ± 10.68	–	–
Duration of Peripheral Neuropathy, years	13.81 ± 4.06	14.31 ± 4.06	–	–
Duration of diabetic foot ulcers, months	3.34 ± 2.25	9.34 ± 2.25	–	–
Gabapentin, g/d	2.95 ± 0.47	1.31 ± 0.38	9.71	<0.001
Smokers, *n* (%)	61 (66.3%)	49 (53.3%)	–	–
VAS, cm	8.38 ± 1.26	2.33 ± 1.30	34.31	<0.001
QOL, score	191.61 ± 24.12	236.22 ± 23.04	−15.14	<0.001

BMI, body mass index; QOL, quality-of-life; VAS, visual analog score; * $\bar{x}$ ± SEM.

### Amputation rate

The amputation rate above the ankle is 3.26% and the amputation rate below the ankle is 5.43%. The overall amputation rate is 8.69%.

### Impact on VAS

The patients’ preoperative pain score was 8.38 (95% CI, 8.12–8.64) cm, while the 6-month postoperative pain score was 2.33 (95% CI, 2.06–2.60) cm (*p* < 0.05), and the clinical response rate was 71.87% (Table 1).

### Impact on QOL

The patients’ preoperative QOL score was 191.61(95% CI, 186.61–196.60), while the 6-month postoperative QOL score was 236.22(95% CI, 231.45–240.99) (*p* < 0.05), and the clinical response rate was 24.74% (Table 1).

### Impact on nerve conduction velocity

**Figure 1:**
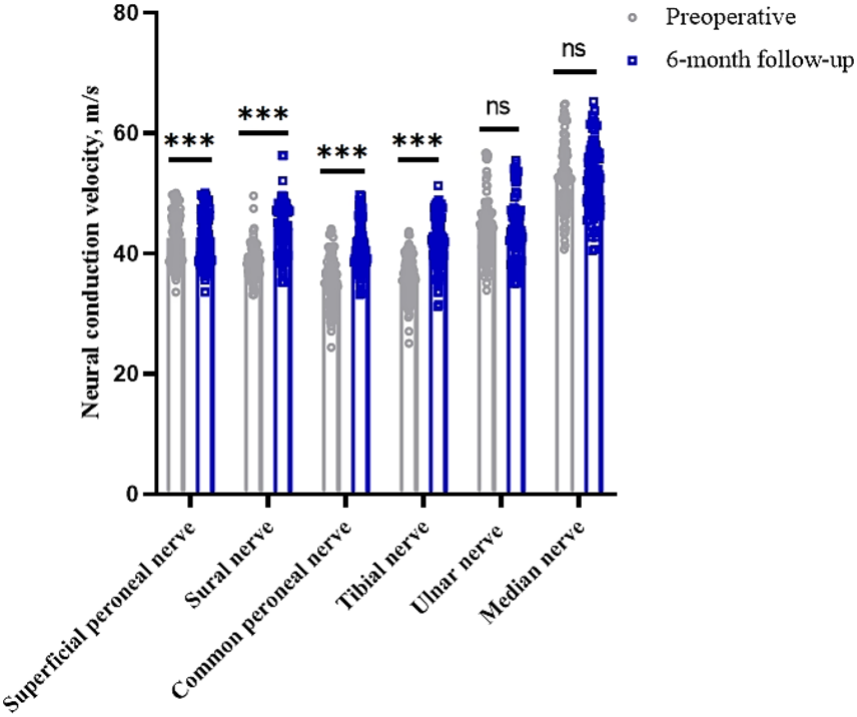
Changes in nerve conduction velocities in peripheral nerves of the limb before and 6 months after surgery. *p < 0.05; **p < 0.01; ***p < 0.001; NS, *p* > 0.05.

The conduction velocities of the common peroneal, sural, superficial peroneal and tibial nerves in the lower limbs of the patients before surgery were 35.51 (95% CI, 34.67–36.35) m/s, 38.61 (95% CI, 38.05–39.18) m/s, 38.21 (95% CI, 37.38–39.04) m/s and 36.11 (95% CI, 35.38–36.85) m/s, respectively. At 6 months after surgery, they were 41.33 (95% CI, 40.46–42.20) m/s, 44.15 (95% CI, 43.26–45.03) m/s, 42.68 (95% CI, 41.82–43.54) m/s and 41.92 (95% CI, 41.06–42.79) m/s, respectively (*p* < 0.05). The clinical remission rates were 17.22, 14.44, 12.22 and 16.58%, respectively. The conduction velocities of the median and ulnar nerves in the upper limbs were 52.62 (95% CI, 51.41–53.78) m/s and 43.87 (95% CI, 42.88–44.98) m/s before surgery and 52.29 (95% CI, 51.11–53.42) m/s and 43.89 (95% CI, 42.87–44.98) m/s at 6 months after surgery (*p* > 0.05), with clinical remission rates of −0.56 and 0.14%, respectively (Table 2 and Figure 1). The Pearson correlation coefficient between VAS and mean nerve conduction velocity was −0.86 before surgery and −0.89 after surgery.

**Table 2:** Changes in nerve conduction velocity, latency and amplitude in peripheral nerves before and 6 months after surgery.

Variable (*n* = 92)		Preoperative*	Postoperative*	CRR	*T* value	*p* values
Common peroneal nerve	Velocity, m/s	35.51 ± 4.06	41.33 ± 4.20	17.22%	−14.27	<0.001
	Latency, ms	20.79 ± 3.74	18.20 ± 3.54	9.56%	4.87	<0.001
	Amplitude, mv	11.00 ± 2.12	14.97 ± 3.47	42.67%	−8.71	<0.001
Tibial nerve	Velocity, m/s	36.11 ± 3.53	41.92 ± 4.18	16.58%	−15.64	<0.001
	Latency, ms	20.72 ± 2.95	16.50 ± 2.84	19.08%	10.82	<0.001
	Amplitude, mv	13.08 ± 3.92	17.08 ± 4.71	44.06%	−6.75	<0.001
Superficial peroneal nerve	Velocity, m/s	38.21 ± 4.00	42.68 ± 4.15	12.22%	−11.81	<0.001
	Latency, ms	3.24 ± 0.81	2.51 ± 0.82	1.90%	5.75	<0.001
	Amplitude, μv	4.27 ± 1.17	6.27 ± 2.40	59.41%	−7.78	<0.001
Sural nerve	Velocity, m/s	38.61 ± 2.72	44.15 ± 4.26	14.44%	−14.60	<0.001
	Latency, ms	2.95 ± 0.85	2.61 ± 0.79	3.95%	2.93	0.006
	Amplitude, μv	7.84 ± 2.16	11.56 ± 3.89	62.24%	−7.67	<0.001
Median nerve	Velocity, m/s	52.62 ± 5.68	52.29 ± 5.56	−0.56%	1.86	0.689
	Latency, ms	11.55 ± 2.01	11.25 ± 1.94	−14.91%	1.10	0.306
	Amplitude, mv	17.65 ± 6.45	16.20 ± 7.10	0.35%	1.81	0.147
Ulnar nerve	Velocity, m/s	43.87 ± 5.07	43.89 ± 5.08	0.14%	0.02	0.995
	Latency, ms	3.07 ± 0.98	3.18 ± 1.03	−0.23%	−0.75	0.458
	Amplitude, μv	7.12 ± 1.82	6.51 ± 3.12	−1.60%	1.65	0.104

CRR, clinical remission rate; * $\bar{x}$ ± SEM.

### Impact on neural conduction latency

**Figure 2:**
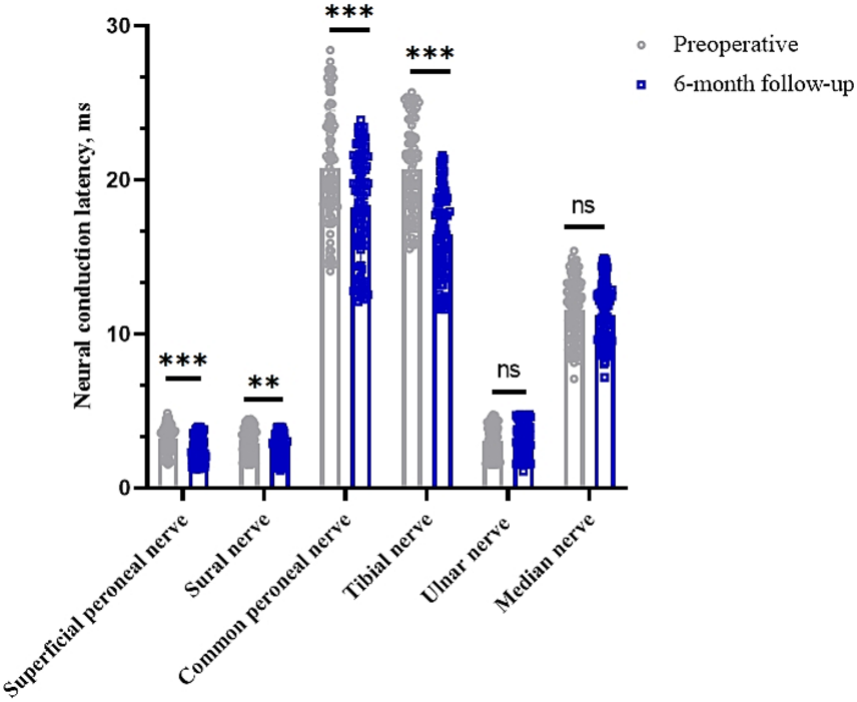
Changes in nerve conduction latency in peripheral nerves of the limb before and 6 months after surgery. *p < 0.05; **p < 0.01; ***p < 0.001; NS, *p* > 0.05.

The latencies of the common peroneal, sural, superficial peroneal and tibial nerves in the lower limbs of the preoperative patients were 20.79 (95% CI, 20.01–21.56) ms, 2.95 (95% CI, 2.77–3.12) ms, 3.24 (95% CI, 3.07–3.40) ms and 20.72 (95% CI, 20.11–21.33) ms, respectively. The postoperative 6 months were 18.20 (95% CI, 17.47–18.93) ms, 2.61 (95% CI, 2.45–2.78) ms, 2.51 (95% CI, 2.34–2.68) ms, and 16.50 (95% CI, 15.91–17.09) ms, respectively (*p* < 0.05). Clinical remission rates were 9.56, 3.95, 1.90, and 19.08%, respectively. The latency of the median and ulnar nerves in the upper limbs before surgery was 11.55 (95% CI, 11.13–11.97) ms and 3.07 (95% CI, 2.87–3.27) ms, respectively, while the latency at 6 months after surgery was 11.25 (95% CI, 10.85–11.65) ms and 3.18 (95% CI, 2.97–3.39) ms (*p* > 0.05), with clinical remission rates of −14.91% and −0.23%, respectively (Table 2 and Figure 2).

### Impact on neural conduction amplitude

**Figure 3:**
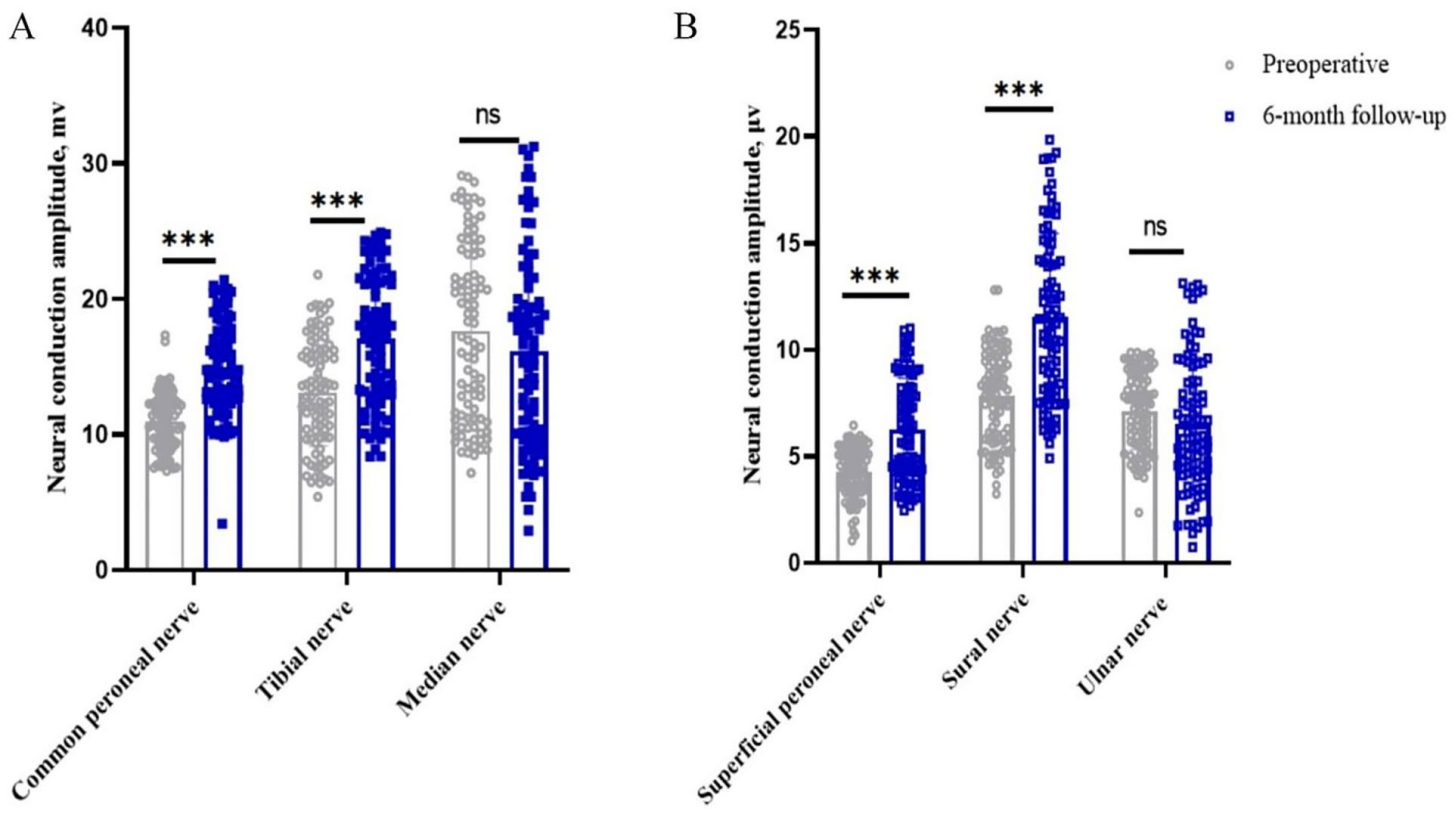
Changes in nerve conduction amplitude in peripheral nerves of the limb before and 6 months after surgery. *p < 0.05; **p < 0.01; ***p < 0.001; NS, *p* > 0.05. **(A)** Changes in peripheral motor nerve conduction amplitude before and 6 months after surgery; **(B)** Changes in peripheral sensory nerve conduction amplitude before and 6 months after surgery.

In the motor nerve, the amplitude of the common peroneal, tibial and median nerves in the patients’ limbs before surgery was 11.00 (95% CI, 10.56–11.44) mv, 13.08 (95% CI, 12.27–13.89) mv and 17.65 (95% CI, 16.32–18.99) mv, respectively. The postoperative 6-month values were 14.97 (95% CI, 14.97–15.69) mv (*p* < 0.05), 17.08 (95% CI, 16.10–18.05) mv (*p* < 0.05), and 16.20 (95% CI, 14.73–17.67) mv (*p* > 0.05), respectively. The clinical remission rates were 42.67, 44.06 and 0.35%, respectively. In the sensory nerves, the preoperative amplitudes of the superficial peroneal, sural and ulnar nerves were 4.27 (95% CI, 4.03–4.52) μv, 7.84 (95% CI, 7.39–8.29) μv and 7.12 (95% CI, 6.75–7.50) μv, respectively. At 6 months after surgery, it was 6.27 (95% CI, 5.78–6.77) μv (*p* < 0.05), 11.56 (95% CI, 10.75–12.36) μv (*p* < 0.05), and 6.51 (95% CI, 5.86–7.16) μv (*p* > 0.05), with clinical remission rates of 59.41, 62.24%, and −1.60%, respectively (Table 2 and Figures 3A,B).

### Impact on vibration perception threshold

**Figure 4:**
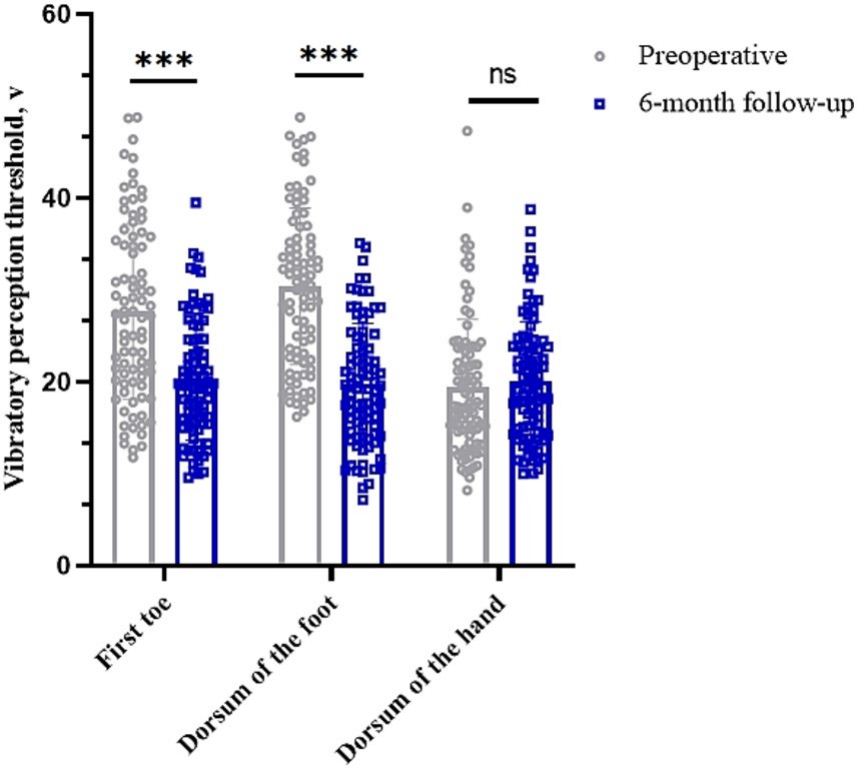
Changes in vibration perception thresholds in different parts of the limb before and 6 months after surgery. *p < 0.05; **p < 0.01; ***p < 0.001; NS, *p* > 0.05.

The vibratory sensory thresholds of the first toe and dorsum of the foot were 27.69 (95% CI, 25.63–29.74) V and 30.40 (95% CI, 28.58–32.22) V preoperatively and 20.17 (95% CI, 18.77–21.57) V and 19.79 (95% CI, 18.39–21.19) V at 6 months postoperatively, respectively (*p* < 0.05). The vibration perception threshold of the dorsum of the hand was 19.47 (95% CI, 17.95–20.98) V preoperatively and 20.13 (95% CI, 18.79–21.46) V 6 months postoperatively (*p* > 0.05) (Table 3 and Figure 4).

**Table 3:** Changes in vibration perception threshold before and 6 months after surgery.

Times	First toe (*n* = 85)	Dorsum of the foot (*n* = 87)	Dorsum of the hand (*n* = 92)
Preoperative*, v	27.69 ± 9.53	30.40 ± 8.53	19.47 ± 7.32
Postoperative*, v	20.17 ± 6.49	19.79 ± 6.58	20.13 ± 6.45
*T* value	7.48	11.49	−0.80
*p* values	<0.001	<0.001	0.426

* $\;\bar{x}$ ± SEM.

## Discussion

### Mechanism of action

The pathophysiology of PDPN is complex, but the mechanisms converge on a unifying theme of bioenergetic failure in the peripheral nerves due to their unique anatomy (36). It is generally accepted that DPN begins with damage to small unmyelinated nerve fibres, followed by damage to small myelinated nerve fibres and, as the disease progresses, damage to large myelinated nerve fibres (37, 38). However, there are no prospective longitudinal studies to confirm or refute this idea.

### Operational procedures of intervention

The aim of this study was to evaluate the efficacy of SCS in the treatment of DPN. A total of 92 people were implanted with permanent stimulators in this study, 2 people withdrew for financial reasons and 3 people were implanted with temporary stimulators that did not work. The results indicate that, for the secondary endpoint, patients’ QOL and VAS scores were significantly improved at 6 months after spinal cord stimulation. The stability of neurophysiological indices (e.g., nerve conduction velocity (NCV) and visual analogue scale (VAS) scores) in the upper limbs suggests that the therapeutic effects of spinal cord stimulation (SCS) are localised rather than systemic. This finding further reinforces the specificity of SCS interventions, given that systemic confounders (e.g., placebo effects or alterations in systemic neuroplasticity) typically manifest in both limbs. Compared to the peripheral nerves of the upper limbs, the conduction velocity, latency and amplitude of the peripheral nerves in the lower limbs of the patients were significantly improved at 6 months after surgery. In addition, vibration perception thresholds in the first toe and dorsum of the foot were significantly improved at 6 months postoperatively. For the primary endpoint, we found that SCS maintained a low amputation rate within 6 months after surgery. This is consistent with our previous study that SCS can relieve lower limb pain, improve conduction velocity, dilate arterial blood vessels and improve quality of life in patients with diabetic foot (26, 27). However, the sample size of this study was significantly larger than previous studies. We not only investigated the effects of SCS on the conduction velocity, latency and amplitude of the lower limb peripheral nerves, including the common peroneal, sural, superficial peroneal and tibial nerves, but also formed a cohort study compared with the upper limb peripheral nerves, including the median and ulnar nerves, making our research results more reliable.

### Comparative analysis with existing therapies

In a randomised clinical trial with 216 participants, although the intervention group reduced VAS by 76.3% at 6 months and 70.3% at 12 months after surgery (39, 40). SCS activates thick Aβ fibres in the dorsal columns of the spinal cord. Excitation of these fibres inhibits the synaptic transmission of pain-related C fibres (nociceptive) and Aδ fibres (allodynia) in the dorsal horn of the spinal cord, thereby closing the ‘pain gate’. At the same time, SCS improves microcirculation by inhibiting sympathetic output and increasing NO (nitric oxide) release (12, 13). Five-year long-term follow-up data suggest that the efficacy of SCS in relieving pain at specific frequencies decreases over time (41). At the same time, SCS can improve the symptoms of severe lower limb ischaemia, promoting wound healing and limb preservation. In a prospective study, transcutaneous partial pressure of oxygen (TcPO2) in the lower limb was significantly improved in more than 70% of patients after SCS implantation (42). In another retrospective study, 97% of patients with severe lower limb ischaemia achieved limb preservation and 73% had healed ulcers 1 year after SCS implantation. Thus, SCS is an effective alternative for patients who are not candidates for endovascular reconstruction (43).

As with any retrospective study, this study has some limitations because it was not a prospective randomised controlled trial, there was no control group in this study, and the follow-up period was relatively short at 6 months. In addition, this study only assessed improvements in pain, quality of life and nerve conduction function and did not further investigate specific mechanisms of action. We will continue to investigate these issues in animal studies in the future.

In conclusion, SCS in the treatment of diabetic peripheral neuropathy can improve lower limb peripheral nerve conduction velocities, reduce latency and increase amplitude, lower vibration perception thresholds and ultimately reduce amputation rates, in addition to its known efficacy in significantly reducing lower limb pain and improving quality of life. Further clinical and basic research should be conducted in the future to support our claims.

## Data Availability

The original contributions presented in the study are included in the article/supplementary material, further inquiries can be directed to the corresponding authors.
